# BioMOF@cellulose Glycerogel Scaffold with Multifold Bioactivity: Perspective in Bone Tissue Repair

**DOI:** 10.3390/gels10100631

**Published:** 2024-09-30

**Authors:** Albert Rosado, Alejandro Borrás, Miguel Sánchez-Soto, Magdaléna Labíková, Hubert Hettegger, Rosa Ana Ramírez-Jiménez, Luís Rojo, Luís García-Fernández, María Rosa Aguilar, Falk Liebner, Ana M. López-Periago, José A. Ayllón, Concepción Domingo

**Affiliations:** 1 Institut de Ciència de Materials de Barcelona (ICMAB), Consejo Superior de Investigaciones Científicas (CSIC), Campus UAB s/n, 08193 Bellaterra, Spain; aborras@icmab.es (A.B.); amlopez@icmab.es (A.M.L.-P.); 2 Departament de Ciència i Enginyeria de Materials, Escola d’Enginyeria de Barcelona Est (EEBE), Universitat Politècnica de Catalunya-Barcelona Tech (UPC), 08019 Barcelona, Spain; m.sanchez-soto@upc.edu; 3 Institute of Chemistry of Renewable Resources, University of Natural Resources and Life Sciences, Vienna (BOKU), Konrad-Lorenz-Strasse 24, A-3430 Tulln an der Donau, Austria; magdalena.labikova@vscht.cz (M.L.); hubert.hettegger@boku.ac.at (H.H.); falk.liebner@boku.ac.at (F.L.); 4 Department of Organic Chemistry, University of Chemistry and Technology, Prague (UCT), Technická 5, 160 00 Praha 6-Dejvice, Czech Republic; 5 Christian Doppler Laboratory for Cellulose High-Tech Materials, University of Natural Resources and Life Sciences, Vienna (BOKU), Konrad-Lorenz-Strasse 24, A-3430 Tulln an der Donau, Austria; 6 Instituto de Ciencia y Tecnología de Polímeros (ICTP-CSIC), C/Juan de la Cierva, 3, 28006 Madrid, Spain; raramirez@ictp.csic.es (R.A.R.-J.); rojodelolmo@ictp.csic.es (L.R.); luis.garcia@csic.es (L.G.-F.); mraguilar@ictp.csic.es (M.R.A.); 7 Networking Biomedical Research Centre in Bioengineering, Biomaterials and Nanomedicine (CIBER-BBN), Av. Monforte de Lemos, 3-5, 28029 Madrid, Spain; 8 Departament de Química, Universitat Autònoma de Barcelona (UAB), Campus UAB s/n, 08193 Bellaterra, Spain; joseantonio.ayllon@uab.es

**Keywords:** glycerogel, cellulose, bioMOF, composite, scaffold, tissue repair

## Abstract

The development of new biomaterials for musculoskeletal tissue repair is currently an important branch in biomedicine research. The approach presented here is centered around the development of a prototypic synthetic glycerogel scaffold for bone regeneration, which simultaneously features therapeutic activity. The main novelty of this work lies in the combination of an open meso and macroporous nanocrystalline cellulose (NCC)-based glycerogel with a fully biocompatible microporous bioMOF system (CaSyr-1) composed of calcium ions and syringic acid. The bioMOF framework is further impregnated with a third bioactive component, i.e., ibuprofen (ibu), to generate a multifold bioactive system. The integrated CaSyr-1(ibu) serves as a reservoir for bioactive compounds delivery, while the NCC scaffold is the proposed matrix for cell ingrowth, proliferation and differentiation. The measured drug delivery profiles, studied in a phosphate-buffered saline solution at 310 K, indicate that the bioactive components are released concurrently with bioMOF dissolution after ca. 30 min following a pseudo-first-order kinetic model. Furthermore, according to the semi-empirical Korsmeyer-Peppas kinetic model, this release is governed by a case-II mechanism, suggesting that the molecular transport is influenced by the relaxation of the NCC matrix. Preliminary in vitro results denote that the initial high concentration of glycerol in the NCC scaffold can be toxic in direct contact with human osteoblasts (HObs). However, when the excess of glycerol is diluted in the system (after the second day of the experiment), the direct and indirect assays confirm full biocompatibility and suitability for HOb proliferation.

## 1. Introduction

Bone-related injuries and diseases are common health disorders that can affect indiscriminately the whole population [1]. On some occasions, when the diagnosis is favorable, the medical treatment relies just on the inherent self-regeneration capacity of bone tissues. However, when massive bone damage or degenerative pathologies occur, invasive clinical surgery is required to achieve a functional restoration. The gold standard in surgical operations consists of the incorporation of human bone tissue, either autograft or allograft, into the damaged zone to fill in large defects [2]. Nevertheless, both approaches present several drawbacks, e.g., bone autographs frequently involve long-term discomfort, infections, and chronic pain, whereas the use of bone allographs depends entirely on the availability of compatible bone tissues in a donor bank, also portraying a potential risk of disease transmission and immune rejection [3]. These limitations have spurred scientists to develop new materials in the field of bone tissue engineering, aiming to reduce morbidity and extend the availability of bone grafts. In this context, synthetic scaffolds have emerged as the most promising alternative to bone grafts [4]. Outstanding research is currently in progress to produce an optimal biomaterial for each scenario [5]. In general terms, to promote osteoconductivity, bone scaffolds must present biocompatibility, adequate mechanical properties to support bone compression, multi-scale micro and macro porosity to allow cell proliferation and angiogenesis, and preferably, degradation simultaneous to bone tissue growth [6].

Synthetic scaffolds can be classified from the point of view of the material as metallic, ceramic, and polymeric [7]. Among them, biopolymeric scaffolds display certain advantages regarding workability and physicochemical properties [8]. From this family, cellulose-based scaffolds are remarkably interesting biomaterials owing to the immense abundance of the raw material, easy conformability, moisture control, and adjustable mechanical and degradation properties via functionalization of the surface hydroxyl groups [9,10,11]. Apart from offering an optimal platform for osteoblast proliferation, advanced scaffolds are currently designed to contain active ingredients, e.g., antibiotic and anti-inflammatory drugs and growth factors, which are locally supplied to the affected area [9,12]. These therapeutic molecules are often delivered encapsulated into porous supports to control their release and prevent their degradation. In this sense, recently developed bioMOFs (biological metal–organic frameworks), assembled from non-toxic naturally occurring biomolecules and biocompatible metal ions, have shown promising applicability as drug carriers owing to their outstanding porosity, versatile surface chemistry, and potential intrinsic pharmaceutical activity [13,14,15,16]. The interesting properties of bioMOFs have opened up a novel area of research in biomaterials science. However, only some examples of biocomposites constituted by MOFs integrated into polymeric scaffolds can be found in the literature [17]. Hence, one of the main objectives of this work is to extend the knowledge in this area by developing new fabrication methods for this type of composites. An additional novelty is based on the use of an original bioMOF, namely CaSyr-1, recently synthesized in our laboratories [18]. Remarkably, it was demonstrated that CaSyr-1 enables the development of triple therapeutic entities, involving bioactive Ca^2+^, syringic acid, and an impregnated drug, which supports the development of therapeutic regimens based on multiple drugs for the treatment of particular diseases [19]. Indeed, calcium-based materials are known to be osteoconductive and, in some cases, osteoinductive [20]. Moreover, syringic acid presents multiple biological activities, including anti-bacterial, anti-inflammatory, and anti-oxidant [21]. In addition, some studies have indicated that syringic acid promotes osteoblasts differentiation [22]. The use of CaSyr-1 in scaffolds has some additional advantages. First, it can be synthesized using a green solvent procedure, thus guaranteeing full biocompatibility. Second, comparing several reported bioMOFs, CaSyr-1 stands out due to its high apparent surface area (>1000 m^2^g^−1^) and large micropore diameter (1.4 nm) that enables the encapsulation of a wide range of active ingredients.

Pursuing new medical applications for CaSyr-1, the main aim of this work is the fabrication of a bioscaffold for bone regeneration, constituted, as the principal components, by nanocrystalline cellulose (NCC) biopolymer supporting CaSyr-1 nanoparticles (NPs). The mesoporosity and macroporosity of the scaffold, necessary for cell growth and formation of blood vessels at the new bone, are generated in an aerogel-like structure within the NCC framework [23]. To this system, the addition of the plasticizer glycerol was essential to produce a material with viable mechanical properties for bone scaffolding [24]. An aerogel charged with glycerol, denoted as glycerogel, was thus synthesized [25]. To fulfill the desired global action of the scaffold, the bioMOF is loaded with ibuprofen (ibu), a drug with anti-inflammatory effects. Preliminary in vitro tests, related to drug delivery and osteoblasts proliferation, are performed to demonstrate the potential scaffold prospective in biomedicine.

## 2. Results and Discussion

### 2.1. Composite Synthesis and Composition

**Figure 1:**
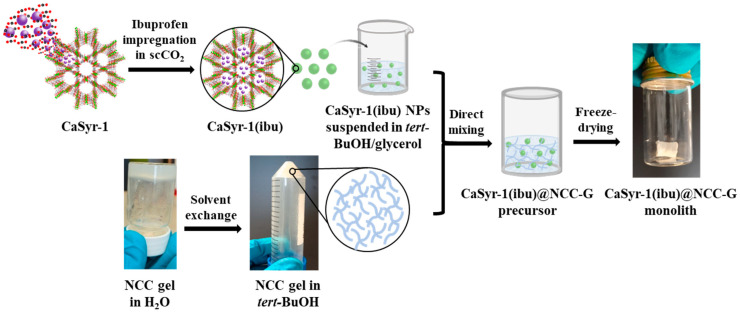
Schematic representation of the main steps followed in the preparation of the target CaSyr-1(ibu)@NCC-G glycerogel.

The fabrication of the targeted composite scaffold with biomedical activity, CaSyr-1(ibu)@NCC-G, involves the individual synthesis of its components (Figure 1). The discontinuous phase in the composite consists of NPs of the bioMOF CaSyr-1 impregnated with ibuprofen. The pristine CaSyr-1 was synthesized following a recently reported green strategy based on the use of ethanol (EtOH) [18]. A supercritical CO_2_ (scCO_2_) method was chosen to encapsulate the ibuprofen drug into the bioMOF micropores, taking advantage of the high solubility of this compound in this fluid (ca. 1.8 × 10^−3^ mole fraction under working conditions) [26] and the high effectiveness of this solvent to penetrate small pores due to its high diffusivity and absence of surface tension [27]. To reach high ibuprofen loadings, the used weight ratio of ibuprofen-to-bioMOF was 1.7:1. The loading of ibuprofen in the CaSyr-1(ibu) sample was estimated by ^1^H-NMR (Figure S1). The ibuprofen signal at δ = 0.86 ppm was integrated with respect to the syringic acid (H_2_Syr) signal at δ = 3.80 ppm, since both signals correspond to the integration of 6 H in the respective pristine organic molecules. The integration of these signals suggests an ibuprofen loading of 41 wt% and a syringic acid content of 46 wt% and, thus, about 9 wt% of calcium, according to the CaSyr-1-simplified empirical formula [Ca(Syr)(H_2_O)].

The used polymeric matrix to support the bioMOF was a cellulosic glycerogel. For the material preparation, NCC particles were first allowed to self-assemble in water at a concentration of 7–8 wt%, yielding a highly viscous hydrogel with multiple hydrogen bonding (H-bond), in which the dispersion of the bioMOF NPs was not feasible. To largely preserve the activated cellulose surface and gel structure while reducing the viscosity, water was gradually replaced with *tert*-butyl alcohol (*tert*-BuOH), a medium with a lower tendency than water to form hydrogen bonds. Additionally, this solvent is suitable for freeze-drying and additionally characterized by minimum acute oral toxicity [28,29]. Nevertheless, the resulting NCC alcogel in *tert*-BuOH lacks the required stability to form mechanically robust aerogels. Hence, to increase the firmness of the product, glycerol was selected as a plasticizer agent for the system. This additive favors interparticle connections in H-bond-assembled gels while reducing intraparticle interactions, with a concomitant increment of the void space, which increases flexibility and decreases the brittleness of the overall structure [30]. A cellulose-to-glycerol weight ratio of 1:4 was selected to prepare the glycerogel, since this proportion gave stable monoliths. It turned out that the dispersion of the bioMOF works best if the CaSyr-1(ibu) NPs are firstly dispersed in a mixture of *tert*-BuOH and glycerol (1:0.25 *v*/*v*) and then the suspension is added to the *tert*-BuOH alcogel containing NCC. Once the CaSyr-1(ibu)@NCC-G composite alcogel was formulated, *tert*-BuOH must be removed. Aiming to preserve both the interconnectivity of the pores and the large specific surface areas, as well as to avoid thermal decomposition, two main strategies, either low-temperature scCO_2_ drying or lyophilization, can be used for this purpose [31,32]. These techniques are the most appropriate to prevent major structural collapse of the gels during drying, maintaining the porous integrity of their solid network structures. Since ibuprofen is highly soluble in scCO_2_, freeze-drying was the method of choice for removing the alcohol, thus circumventing the potential obstacle of drug removal during scCO_2_ drying. Following this approach, CaSyr-1(ibu)@NCC-G glycerogels were obtained as cylindrical white sticky monoliths with negligible shrinkage (Figure 1). According to ICP-MS, the mass ratio of CaSyr-1(ibu) with respect to NCC in the matrix was ca. 1:2.7, which is equivalent to about 6 wt% related to the mass of the CaSyr-1(ibu)@NCC-G composite, while NCC and glycerol account for 16 wt% and 78 wt%, respectively.

### 2.2. Scaffold Structure and Porosity

**Figure 2:**
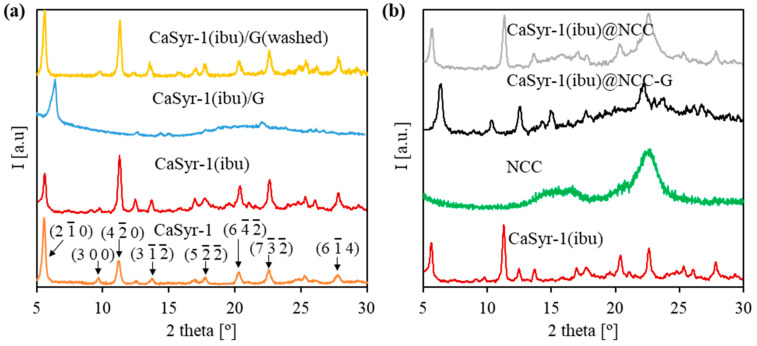
XRD patterns of the prepared samples: (**a**) CaSyr-1 NPs with different additives (including CaSyr-1 planes), and (**b**) target composite and individual components.

**Figure 3:**
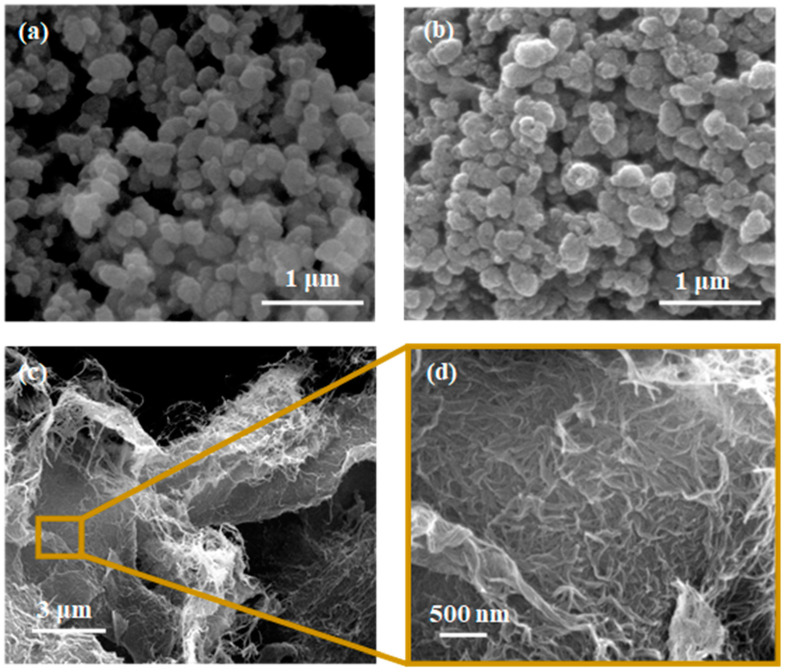
SEM images of the individual components in the composite: (**a**) CaSyr-1 NPs, (**b**) CaSyr-1(ibu) NPs, and (**c**,**d**) NCC at different magnifications.

**Figure 4:**
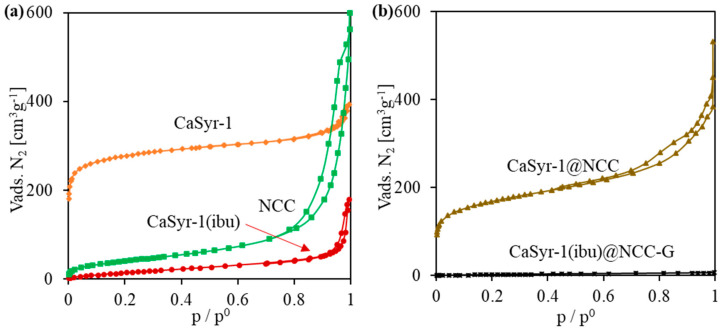
N_2_ adsorption/desorption measurements of the prepared samples: (**a**) individual components of the composite, and (**b**) aerogels with and without additives.

The structure and porosity of the designed composite strongly rely on the characteristics provided by its main components, i.e., the CaSyr-1(ibu) NPs and cellulosic cell matrix. The bioMOF CaSyr-1 has been described as a highly crystalline solid with the most intense XRD lines at 2ϴ = 5.6 and 11.5° (Figure 2a) [18]. This bioMOF displays a highly microporous lattice, with channels of 1.4 nm in diameter, bulky enough to encapsulate a wide range of bioactive compounds, including the anti-inflammatory ibuprofen drug, which has spatial molecular dimensions of ca. 1.03–1.30 × 0.34–0.52 nm, depending on the molecular arrangement in space [33]. For the CaSyr-1(ibu) sample, the presence of ibuprofen in the pores did not involve a shift of the XRD peaks of CaSyr-1 but only some variability in the relative intensity of the main signals (Figure 2a). It has been previously established that the intensity of the peak at 5.6° increases for highly activated samples and decreases when the micropores become less accessible [18]. Regardless, even at the high ibuprofen loading of ca. 40 wt%, attained using scCO_2_ as a carrier solvent, the main reflections of recrystallized ibuprofen particles forming part of the bulk of the sample, envisaged at 2ϴ = 6.1, 16.6 and 20.3°, were not noticeable in the XRD pattern. SEM images indicated that the morphology of bioMOF NPs before and after ibuprofen impregnation was similar (Figure 3a,b). Typical ibuprofen needles were not displayed in the loaded CaSyr-1(ibu) sample, confirming the absence of the recrystallized drug (Figure 3b). N_2_ adsorption–desorption analysis revealed a type I isotherm for pristine CaSyr-1 NPs, characteristic of a microporous material (Figure 4a), with an apparent BET surface area > 1000 m^2^g^−1^. Compared to that, the volumetric adsorption of N_2_ into the micropores of CaSyr-1(ibu) suffered a complete drop, since the open volume was mainly occupied by ibuprofen molecules (Figure 4a). For the as-synthesized and impregnated CaSyr-1, some mesoporosity could be observed in the isotherms represented by hysteresis at high relative pressures, ascribed to NPs aggregation. For sample CaSyr-1(ibu), the drop in microporosity, the absence of ibuprofen diffraction peaks, the reduced intensity of the signal at 5.6°, and the lack of elongated crystals are all considered strong pieces of evidence for the drug being molecularly impregnated inside the bioMOF micropores and not precipitated as crystals in the bulk. This is the typical result described for scCO_2_ impregnation processes [27]. Hence, it is assumed that, in the CaSyr-1(ibu) sample, ibuprofen is molecularly dispersed throughout the interconnected system of micropores and homogeneously distributed across the large pore volume of the bioMOF.

Embedding of CaSyr-1(ibu) NPs in the open-porous water-free polymeric matrix of nanocrystalline cellulose to obtain the composite CaSyr-1(ibu)@NCC-G was accomplished by dispersing the bioMOF in a mixture of *tert*-BuOH and glycerol. Therefore, to start with the analyses, the possible structural interactions between CaSyr-1(ibu) and glycerol in a solid were studied. For that, the two components were mixed in *tert*-BuOH, which was further evaporated, leading to sample CaSyr-1(ibu)/G. XRD analysis indicated that the addition of glycerol modified the structure of CaSyr-1 (Figure 2a). The main peak observed for CaSyr-1 at 2ϴ = 5.6° was shifted by ca. 0.8° towards higher values, i.e., 2ϴ = 6.4°. Actually, structural modifications caused by external agents (additives, temperature, pressure, pH, etc.) are a common feature observed in MOFs with certain structural flexibility, which concomitantly leads to alteration of the respective diffraction patterns [34]. In this work, distortion of the structure of the flexible CaSyr-1 bioMOF was induced by the highly viscous glycerol, as it presumably exerts considerable force on the walls of the framework, either from inside or, most probably, outside the pores [35]. The framework deformation, though, was reversible, which was demonstrated by extracting the glycerol additive from the CaSyr-1(ibu)/G sample with ethanol. The XRD pattern of the washed sample CaSyr-1(ibu)/G(washed) matched that of the CaSyr-1 NPs (Figure 2a).

The NCC matrix forms the foundation of the designed composite. Hence, a preliminary study of the net NCC freeze-dried structure was performed. The obtained NCC aerogel exhibited the typical broad XRD bands of nanocrystalline cellulose at 2ϴ = 16.5 and 22.5° (Figure 2b) [36]. As observed in the SEM (Figure 3c,d) and TEM images (Figure S2), the solid network consists of loosely assembled two-dimensional flakes of aggregated NCC nanowhiskers. These solid components build up a hierarchical architecture that hosts both meso and macropores. The presence of porosity was further confirmed by N_2_ adsorption–desorption experiments, which revealed a type IV isotherm at medium relative pressures, with H3-type hysteresis attributed to plate-like particles aggregation, and a type II isotherm at high relative pressures characteristic of mesoporous and macroporous structures, respectively (Figure 4a).

**Figure 5:**
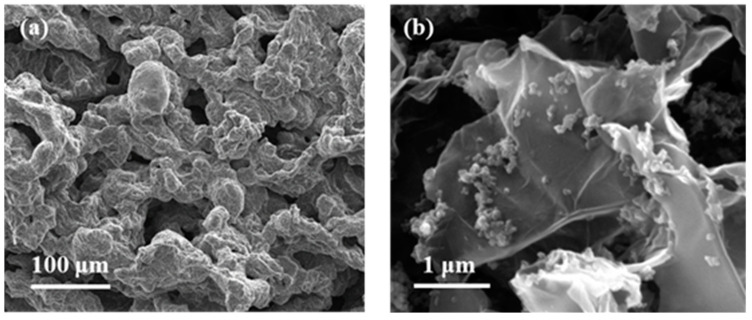
SEM images of the target CaSyr-1(ibu)@NCC-G aerogel: (**a**) cross-section and (**b**) zoom showing the deposited NPs of the bioMOF.

Further, structural changes were observed when mixing the two components, CaSyr-1(ibu) NPs and the NCC gel, and adding the glycerol additive to shape the CaSyr-1(ibu)@NCC-G glycerogel. The CaSyr-1 structure was modified as described for the CaSyr-1(ibu)/G system, with a shift in the main peaks of CaSyr-1 towards higher values of 2ϴ (Figure 2b). Similar to CaSyr-1(ibu)/G, this effect was attributed to the interaction of glycerol with the framework of the microporous bioMOF. Indeed, a similar composite prepared just as CaSyr-1(ibu)@NCC-G but lacking glycerol did not suffer any noticeable framework distortion (Figure 2b, sample CaSyr-1(ibu)@NCC). The SEM images of the cross-section area of the CaSyr-1(ibu)@NCC-G sample displayed a continuous framework with interconnected macropores (Figure 5a). A close view showed that CaSyr-1(ibu) NPs were present lying on the surface of the aggregated NCC flakes (Figure 5b). The physisorbed volume of N_2_ was practically zero for the CaSyr-1(ibu)@NCC-G sample (Figure 4b), denoting the absence of micro and mesoporosity, which is explained by ibuprofen filling the micropores of CaSyr-1 and glycerol plugging the mesopores in the NCC matrix. Note that a composite shaped with CaSyr-1 and NCC (sample CaSyr-1@NCC) displayed significant porosity, including both micro and mesoporosity (Figure 4b).

### 2.3. Mechanical Properties

The mechanical properties of the matrix would largely affect the tissue scaffolding potential, with a vital role in bone repair. As previously mentioned, the addition of an appropriate volume of glycerol to the NCC matrix was experimentally proved to be indispensable to produce a material with viable mechanical properties to support the necessary loading for bone scaffolding, the proposed application for CaSyr-1(ibu)@NCC-G. The mechanical properties were studied for this composite by performing cyclic compression tests (Figure S3). CaSyr-1(ibu)@NCC-G exhibits a linear region up to ca. 20% strain, which denotes complete elasticity until this value. From this point, the plastic region is extended to ca. 40% strain—after which, non-reversible densification occurs. According to the collected data, the compressive Young’s modulus, calculated from the slope of the linear elastic region, was 16.8 ± 2.4 kPa, and the maximum compressive strength, calculated at 40% strain, was 5.4 ± 0.8 kPa. The scaffolds prepared in this study are believed to be robust enough and, with sufficient flexibility of the struts surrounding the pores, to carry stem cells and to facilitate ingrowth and proliferation, as well as osteogenic differentiation in bone tissue, a complex material with regions of enormous mechanical heterogeneity [37,38]. The CaSyr-1(ibu)@NCC-G composite was prepared as a foamed monolith with certain flexibility and malleability, attributed to the plasticizing properties of glycerol, which allows the easy form-filling of bone defects. This type of composite is considered acceptable to fulfill the challenges of the implantation of scaffolds in small bone defects with irregular shapes and through minimally invasive surgery [39,40]. For critical-sized load-bearing bone defect reconstruction, synthetic polymeric composites and bio-ceramics are scaffolds with more suitable mechanical properties [6]. Noteworthy, one of the main advantages of nanocellulose-based biopolymeric scaffolds is that the mechanical properties can be easily improved by chemical interaction with glycerol or other additives [23].

### 2.4. Release of Bioactive Compounds

**Figure 6:**
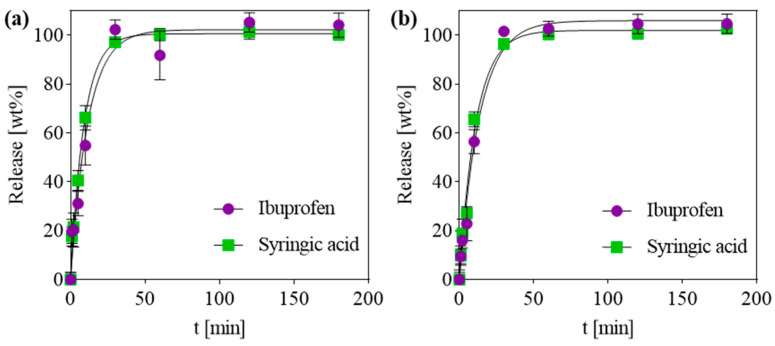
Release profiles of ibuprofen and syringic acid in PBS at 310 K from (**a**) CaSyr-1(ibu) NPs and (**b**) CaSyr-1(ibu)@ NCC-G glycerogel. Fitted lines are obtained by using the PFO model.

Apart from the insertion of a biocompatible scaffolding material, bone regeneration treatments typically require a local dosage of active ingredients, ranging from growth factors to antibiotic and anti-inflammatory drugs [41]. In this work, the CaSyr-1(ibu)@NCC-G composite was tested as a carrier matrix for the local delivery of bioactive compounds. Ibuprofen was used as a model anti-inflammatory drug to study the release properties of active agents from the pores of the bioMOF after embedding the latter in a macroporous cellulosic glycerogel matrix. In vitro tests were performed in PBS at 310 K to ascertain the drug release profiles of syringic acid and ibuprofen from CaSyr-1(ibu)@NCC-G, using the control CaSyr-1(ibu) for comparison. In these particular conditions, the release of the bioactive compounds from CaSyr-1 is promoted by the disintegration of the framework due to the displacement of the coordinated ligand by the formation of calcium phosphates. HPLC analysis of the PBS extracts from CaSyr-1(ibu) and CaSyr-1(ibu)@NCC-G revealed similar release profiles for syringic acid and ibuprofen, reaching a plateau of the maximum release after ca. 30 min (Figure 6a,b). The release data for ibuprofen and syringic acid in both studied samples could be fitted with the equation of the pseudo-first-order (PFO) kinetic model (Q_t_/Q_∞_ = 1 − e^−kPFO·t^, where Q_t_/Q_∞_ is the fraction of drug released at time t and k_PFO_ is the release rate constant). This model has been often applied to represent the release profile of drugs encapsulated in biodegradable polymers [42,43]. To assess any diffusion effect of the NCC-G matrix in the release, the recorded data points up to ca. 60 wt% of the drug release were fitted by applying the semi-empirical Korsmeyer-Peppas kinetic model: Q_t_/Q_∞_ = k_KP_t^n^, where k_KP_ is the release rate constant and n is the release exponent (Figure S4) [44]. The exponent n provides information on the mechanism that governs the release kinetics, and three different processes can be classified according to this value, i.e., Fickian (n ≤ 0.45), case-II (n ≈ 0.89), and anomalous non-Fickian (0.45 < n < 0.89) [45]. In brief, the Fickian diffusional release indicates molecular diffusion of a drug due to a concentration gradient, the case-II relaxational release is drug transport associated with stresses and state transition in hydrophilic polymers that swell in biological fluids, and the anomalous (non-Fickian) diffusion is caused by both Fickian diffusion and case-II transport [46]. For the CaSyr-1(ibu) powder, the n values for syringic acid and ibuprofen release were 0.58 and 0.55, respectively (Figure S4a,b). Hence, the release was driven by anomalous diffusion, with contributions of the concentration gradient and matrix decomposition. The case-II transport effect was much more evident in the syringic acid and ibuprofen release from CaSyr-1(ibu)@NCC-G glycerogel, as reflected in the calculated n values of ca. 0.9 for both compounds (Figure S4c,d). The ibuprofen and syringic acid release mechanism were thus somehow influenced by the relaxation of the polymeric cellulose-based architecture upon water penetration in the system. In any case, the designed scaffold can be used to locally deliver the necessary initial burst dose of drugs with very low solubility in water, like ibuprofen, to cause a rapid onset of the pharmacological effect.

### 2.5. In Vitro Tests

**Figure 7:**
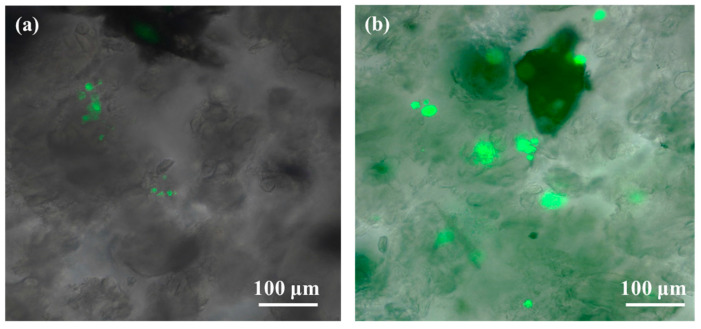
Calcein in vivo staining of HOb after (**a**) 24 and (**b**) 48 h of culture in CaSyr-1@NCC-G.

**Figure 8:**
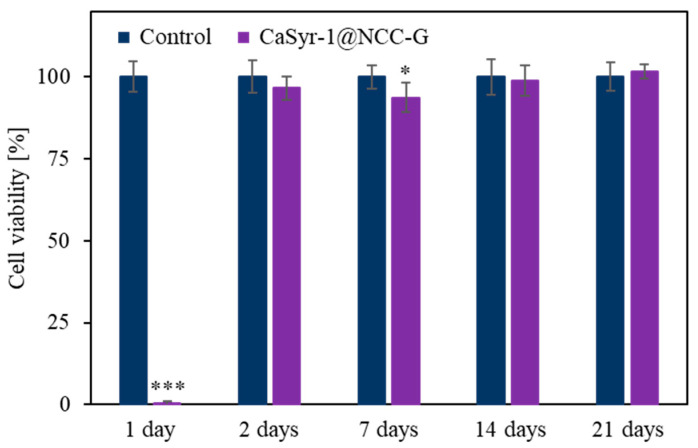
Cell viability of HOb in CaSyr-1@NCC-G leachates compared to the control. Significant differences with the control at each time are marked with * (* *p* < 0.05 and *** *p* < 0.001).

To evaluate the cytotoxicity of the developed scaffold, a sample without ibuprofen, namely CaSyr-1@NCC-G, was used. The drug was not incorporated into the composite to avoid misleading results, as it can be chosen from a wide ranging set of active components, each one with a specific side effect. Initially, a direct assay was chosen to ascertain the toxicity of the released components from the composite. For that, concentrated solutions of human osteoblasts (HObs) were directly cultured on CaSyr-1@NCC-G and observed upon Calcein in vivo staining. In the images captured after 24 h, just little fluorescent spots could be discerned in the scaffolding material, denoting a low concentration of living cells (Figure 7a). Nevertheless, in the subsequent samples, obtained after two days and longer periods, the fluorescence signals exponentially increased (Figure 7b). This particular behavior was cross-checked in an indirect assay consisting of collecting scaffold lixiviates in cell growth medium at different times, which were further cultured with HOb cells. Again, during the first day, the scaffold released a component that damaged the cells, although, for the rest of the assay, cell viability was as high as the one of the controls (Figure 8). These preliminary results are thus analyzed on the basis of scaffold toxicity during the first step of direct and indirect assays. From all the scaffold components, both CaSyr-1 and NCC have previously demonstrated null toxicity for cells in similar assays [18,47]. Consequently, the effect of glycerol was further scrutinized as the origin of toxicity. Glycerol is a sugar alcohol with very low acute oral and dermal toxicity, and it is widely used in a variety of applications in pharmaceuticals, personal care, and food products [48]. In principle, glycerol exhibits full biocompatibility and could be freely used as either a bioactive entity or additive in the formulation of medical stocks without any toxicological concern. Particularly, glycerol has been extensively used as an additive for the assembly of gelatin hydrogels in flexible tissue engineering and synthetic bone scaffolds [49,50]. Nevertheless, it has already been demonstrated that glycerol in culture medium results in significant suppression of the replication of different cell lines, the restraint being dose-dependent [51]. In addition, glycerol has been shown to have harmful osmotic effects on cell membranes at high concentrations and/or high rates of addition [52,53]. These damaging effects also appear in the tests performed in this work during the first hours of cell adaptation at concentrated doses of glycerol (i.e., 0.05–1 wt%). In fact, glycerol is found in the current scaffolding composite in a significantly higher weight ratio than the other components (ca. 80 wt%) and possibly surpasses safe concentration limits just after addition. Actually, in the lixiviated sample taken after 24 h in the indirect assay, glycerol excess could be identified by an optical microscope as birefringent floating drops in the seeded cellular culture medium (Figure S5). These glycerol drops are formed due to the large difference in viscosity between the humectant and the buffer aqueous phase (ca. 0.3 vs. 0.7 × 10^−3^ Pa·s, respectively, at 310 K), also displaying the hydration shell of intermediate water oriented around glycerol-bound water [54]. At diluted glycerol concentrations, as those found in the lixiviates taken after two days and subsequently, glycerol drops were not observed, and the cell damage was ameliorated, likely because the osmotic stress was lessened. Indeed, the recovery of cell proliferation was complete at day two. Collectively, these results indicate that the drawback of initial glycerol-mediated cell growth inhibition must be eliminated before the developed CaSyr-1@NCC-G scaffold meets the required characteristics to enable tissue regeneration. This objective could be achieved by washing the scaffolding material to partially remove glycerol, e.g., by controlled immersion of the composite in a biocompatible solvent in which CaSyr-1 does not get dissolved, such as ethanol or dimethyl sulfoxide, thus leading to a final concentration that did not affect the viability of the cells yet preserved the porous macrostructure of the composite. Even glycerol is miscible with these solvents, and the merging would be a slow process due to the high viscosity of the former [55]. In any case, this strategy could compromise the structural stability of the framework, since glycerol acts as a reinforcement for the NCC matrix. Incorporating other biocompatible plasticizers while decreasing the initial glycerol content could be an alternative approach to reduce the toxicity associated with glycerol, still preserving the physicochemical characteristics of the material.

## 3. Conclusions

This study illustrates the potential of CaSyr-1 bioMOF integrated into nanocrystalline cellulose scaffold for tissue engineering. The tested composite CaSyr-1(ibu)@NCC-G holds promise as the homogeneous microporosity provided by the bioMOF ensures suitable drug release profiles, while the open meso and macro porosity of the NCC matrix is appropriate for angiogenesis and cell proliferation. Nevertheless, pristine nanocrystalline cellulose aerogels lack the necessary structural stability to be used as scaffolds, which is solved here by using viscous glycerol as the additive. The malleable CaSyr-1(ibu)@NCC-G glycerogel would allow the easy form-filling of bone defects, simultaneously providing support for cells. The specific benefits obtained by implementing bioMOFs as a functional component of the cell scaffolding material are demonstrated through the triply bioactivity of CaSyr-1, impregnated inside its cavities with ibuprofen, which dissolves in the presence of simulated body fluids. The release of the bioactive ingredients was boosted by the degradation of the bioMOF framework, which results in a simultaneous release of calcium, syringic acid, and ibuprofen, governed in the composite by a case-II release mechanism, affected by the swelling and relaxation of the NCC-G polymeric matrix. In vitro preliminary tests proved that the as-synthesized composites have an exceeding content of glycerol that inhibits initial HOb proliferation. However, when the glycerol content is diluted in the system, the polymeric scaffold presents full biocompatibility and suitability for HOb proliferation. This initial drawback could be solved by controlled immersion of the scaffold in a proper solvent for glycerol before using it, although this strategy could compromise the structural stability of the material. Other alternatives could focus on reducing the initial content of glycerol while adding another biocompatible plasticizer in order to achieve similar physicochemical properties. Overall, this critical parameter must be addressed before the implementation of the synthetic scaffold in biomedicine.

## 4. Materials and Methods

### 4.1. Materials

The reactants for CaSyr-1 bioMOF synthesis were syringic acid (H_2_Syr; 98%, abcr, Karlsruhe, Germany) and calcium acetylacetonate (Ca(acac)_2_; 98%, abcr, Karlsruhe, Germany), using absolute ethanol (EtOH, Scharlab, Sentmenat, Spain) as a solvent. For drug impregnation, the active compound (*S*)-(+)-ibuprofen (ibu; 99%, Sigma-Aldrich, St. Louis, MO, USA) was dissolved in compressed CO_2_ (99.95 wt%, Carburos Metálicos S.A., Cornellà de Llobregat, Spain). The CaSyr-1(ibu)@NCC-G composite was prepared using NCC (CelluForce Inc., Montreal, QC, Canada), by using water, *tert*-butyl alcohol (*tert*-BuOH; 99%, TCI, Tokyo, Japan), and glycerol (G; 99%, abcr, Karlsruhe, Germany) as solvent, compatibilizer, and swelling agent, respectively. 

### 4.2. Synthetic Procedure

The synthesis of CaSyr-1(ibu)@NCC-G was accomplished by co-homogenization of a CaSyr-1(ibu) dispersion in *tert*-BuOH/glycerol with a NCC gel in *tert*-BuOH, followed by freeze-drying (Figure 1).

*CaSyr-1(ibu) nanopowder.* CaSyr-1(ibu) was prepared at room temperature as previously reported [18]. In short, a dispersion and a solution of 238 mg of Ca(acac)_2_ and 198 mg of H_2_Syr in EtOH (1.0 mmol each), respectively, were sonicated for 1 min before mixing them in a vial, thus precipitating the CaSyr-1 phase by the quantitative consumption of Ca(acac)_2_. The resulting suspension was washed thrice with EtOH by centrifugation, filtered, and dried in a desiccator. The recovered CaSyr-1 nanopowder was activated at 393 K under vacuum (ca. 15 Pa) before impregnation with ibuprofen following a supercritical CO_2_ (scCO_2_) method. In a typical impregnation experiment, a stirred (500 rpm) 100 mL high-pressure vessel was loaded with 250 mg of ibu (1.2 mmol, higher than described in the original protocol) and 150 mg of activated CaSyr-1 powder (0.6 mmol) and pressurized up to 15 MPa (Teledyne Isco 260D pump, Teledyne Isco, Lincoln, NE, USA), maintaining the temperature at 313 K for a period of 20 h. Then, 120 mg of the recovered CaSyr-1(ibu) sample were redispersed in 3.75 mL of a mixture of *tert*-BuOH and glycerol (1:0.25 *v*/*v*).

*NCC gel.* The preparation of the matrix of the composite started with the generation of a NCC gel in water, obtained by dispersing 240 mg of NCC in 3 mL of water. The mixture was homogenized by mechanical stirring and left to rest for 10 min. Then, *tert*-BuOH (7 mL) was added to the gel and mixed by Vortex agitation. The water in the water/*tert*-BuOH mixture was exchanged to *tert*-BuOH following a stepwise removal protocol aided by centrifugation, which consisted of the extraction of 1/4, 1/2, and 3/4 parts of the liquid phase and the addition of the corresponding amount of *tert*-BuOH to maintain the volume constant at 10 mL. The gelled mixture was further washed twice with fresh *tert*-BuOH, discarding the supernatant. 

*CaSyr-1(ibu)@NCC-G composite.* To prepare the CaSyr-1(ibu)@NCC-G composite, the CaSyr-1(ibu) suspension in *tert*-BuOH/glycerol (3.75 mL) was dropwise poured into the vial containing the NCC gel in *tert*-BuOH. The mixture was homogenized by Vortex agitation and mechanical stirring. The content was divided into equal parts of ca. 1 mL and transferred to cylindrical Teflon molds of 1 cm^3^ that were placed into a freezer at 193 K for 1 h. The samples were then lyophilized at about 5–6 Pa using an Alpha 3–4 LSCbasic laboratory freeze-dryer (Martin Christ Gefriertrocknungsanlagen GmbH, Osterode, Germany). The product was obtained as sticky white monoliths of ca. 1 cm^3^.

### 4.3. Characterization

#### 4.3.1. Physicochemical, Morphological, and Mechanical Properties

Proton nuclear magnetic resonance (^1^H-NMR, Bruker Avance NEO 300 MHz, Bruker Corporation, Billerica, MA, USA) was used to quantify the molar ratio of ibu with respect to H_2_Syr in the impregnated sample CaSyr-1(ibu). The analysis was carried out in dimethyl sulfoxide-d_6_ (DMSO-d_6_; 99.5%D, abcr) after digesting the sample in hydrofluoric acid (HF; 40 v% in water, Fluka, Darmstadt, Germany) following a well-established protocol [18]. The ibu signal at δ = 0.86 ppm was integrated with respect to the H_2_Syr signal at δ = 3.80 ppm, since both signals correspond to the integration of 6 H in the respective pristine organic molecules (Figure S1). Inductively coupled plasma mass spectrometry (ICP-MS, 7700x Agilent, Agilent Technologies, Santa Clara, CA, USA) was used to quantify Ca^2+^ in the composite. The samples were digested with a mixture of hydrochloric acid and nitric acid (3:1 *v*/*v*) in a microwave oven previous to the analysis. Powder X-ray diffraction (PXRD, Siemens D5000, Siemens AG, Munich, Germany) patterns of the different materials were recorded at room temperature using the Cu K_α_ incident radiation in the 2ϴ range 5–30° with steps of 0.02° s^−1^. Scanning electron microscopy (SEM, Quanta FEI 200 FEG-ESEM, Thermo Fisher Scientific, Waltham, MA, USA) and transmission electron microscopy (TEM, JEOL 1210, JEOL Ltd., Tokyo, Japan) were utilized to evaluate the morphology of the studied systems. N_2_ physisorption at 77 K (ASAP 2020 Micromeritics, Micromeritics Instrument Corporation, Norcross, GA, USA) was applied to estimate the porosity of the composite and its components. Samples were previously activated at 393 K under vacuum. The specific surface area was calculated by applying the Brunauer–Emmet–Teller (BET) equation to the recorded N_2_ isotherms. The mechanical resistance of CaSyr-1(ibu)@NCC-G to compression was studied in a universal testing machine (Galdabini SUN 2500, Galdabini S.p.A., Busto Arsizio, Italy) equipped with a 1 kN load cell and operating at 1 mm min^−1^ crosshead speed. The material was submitted to semi-static stress pulses to accomplish a 10% strain with respect to the initial length. Afterward, the compressive stress was set to zero before the process was repeated in cumulative cycles with additional strain increments of 10% until the visual cracking of the monolith. The stress (σ) vs. strain (ε) curve was plotted to assess the mechanical properties of the material by determining the elastic, plastic, and densified regions. The compressive Young’s modulus (E) was calculated from the slope of the elastic region: σ = E·ε. The mechanical test was conducted in duplicate (*n* = 2).

#### 4.3.2. Release of Bioactive Compounds

The release of the organic bioactive compounds, i.e., syringic acid and ibuprofen, from the studied composite CaSyr-1(ibu)@NCC-G, and CaSyr-1(ibu) for comparison, in phosphate-buffered saline (PBS) solution was measured by high-performance liquid chromatography (HPLC, Agilent 1100, Agilent Technologies, Santa Clara, CA, USA, equipped with a Chiralpak^®^ QD-AX column (150 × 3 mm, 3 µm particle size, DAICEL Chiral Technologies Europe SAS, Chalon-sur-Saône, France)). The mobile phase was a mixture of methanol/acetic acid/ammonium acetate (MeOH/AcOH/AA; 100/2/0.5 m/m/m), applied with a 0.3 mL min^−1^ flow rate. Measurements were performed in the presence of the internal standard 3,5-dinitrobenzoic acid (DNBA). The observed retention times for ibu, H_2_Syr, and DNBA were 4.3, 4.9, and 12.5 min, respectively, with enough resolution to afford reliable results (Figure S6). Calibration curves were first obtained and displayed as A_X_/A_DNBA_ vs. C_X_/C_DNBA_, where A is the area, C is the concentration, and X refers to the specific bioactive compound (Figure S7). For analyte concentration measurement, a weighted amount of sample, either 65 mg of CaSyr-1(ibu) powder or 175 mg of CaSyr-1(ibu)@NCC-G monolith, was immersed in 50 mL of PBS (pH = 7.4) at 310 K. Flasks were immediately placed into a water shaker system set at the same temperature with the oscillation mode fixed at 100 rpm. Aliquots of 1 mL were withdrawn after specific periods. Then, the DNBA solution in PBS (100 μL, 1 mg mL^−1^) was added to each aliquot, and the obtained mixtures were frozen at 193 K and dried under vacuum. The resulting crudes were subsequently dissolved in 1 mL of the mobile phase and passed through PTFE syringe filters (0.45 µm) prior to the analysis. The concentration of the bioactive compound in each aliquot was calculated by extrapolating the experimental A_X_ signal in the resulting equation, considering the dilution factors. Each aliquot was measured in triplicate (n = 3), and the results were plotted as mean values ± standard deviation.

#### 4.3.3. In Vitro Tests

A sample of CaSyr-1@NCC-G was employed to evaluate the cytotoxicity and cell proliferation capability of the scaffold in fetal human osteoblasts, obtained from the European Collection of Cell Cultures (HObs, 406-05f, ECACC, Salisbury, UK). This sample was used instead of CaSyr-1(ibu)@NCC-G to separately characterize the toxic effects of the composite from the model drug ibuprofen. Prior to the tests, the sample was sterilized at 413 K for 5 h. To evaluate the cytotoxicity, the CaSyr-1@NCC-G sample was submitted to an indirect assay, in which 15 mg of the scaffold was immersed in 3 mL of HOb growth medium (Cell Applications, Inc., Cat. No. 417-500, San Diego, CA, USA) at 310 K, attaining a concentration of 5 mg mL^−1^. Scaffold lixiviates were collected after 1, 2, 7, 14, and 21 days and stored at 253 K before cell culturing. After each extraction, the plate was refilled with fresh medium. Once the last extraction was collected, the HOb cells were cultured in a 96-well plate at 100,000 cells mL^−1^ and incubated at 310 K with 5 v% CO_2_ and 95% humidity for 24 h. Next, the collected extracts were added to the cultured cells and incubated again for 24 h. After this time, the supernatants were removed, and Alamar Blue (10 v% without phenol red, DMEM Gibco Cat. No. 21063-029, Thermo Fisher Scientific, Waltham, MA, USA) was added to the culture, followed by 3 h of incubation. The fluorescence was measured at 560 nm in the plate lector of Synergy HT equipment (Agilent Technologies, Santa Clara, CA, USA). The analyses were performed in triplicate, and the results were represented as the average with the corresponding standard deviation. Separately, a direct assay was also performed to assess the suitability of the scaffold for the adhesion and proliferation of osteoblasts. For that, 50 µL of a concentrated solution of HObs (70,000 cells mL^−1^) was added to fractions of the CaSyr-1@NCC-G scaffold. The samples were submitted to Calcein in vivo staining after different times and observed by optic microscopy. The samples were tested in triplicate with independent extracts (n = 6) and normalized to the control samples. The results were expressed as the mean values ± standard deviation. Statistical analysis was performed using two-way analysis of variance (ANOVA), and the Bonferroni post hoc test was used to determine statistically significant differences between the control and samples at different times (* *p* < 0.05, ** *p* < 0.01, and *** *p* < 0.005).

## Data Availability

The original contributions presented in the study are included in the article/Supplementary Materials, further inquiries can be directed to the corresponding author/s.

## Supplementary Materials

The following supporting information can be downloaded at https://www.mdpi.com/article/10.3390/gels10100631/s1, Figure S1: ^1^H-NMR of CaSyr-1(ibu) after HF treatment and filtration; Figure S2: TEM image of net NCC; Figure S3: Compressive stress-strain curve for CaSyr-1(ibu)@NCC-G; Figure S4: Drug release data fitted with the Korsmeyer-Peppas model; Figure S5: Optical image of glycerol drops in CaSyr-1@NCC-G leachate; Figure S6: Retention times for ibu, H2Syr and DNBA; Figure S7: Calibration curves for HPLC measurements.
