# Supplementary material for: BioMOF@cellulose Glycerogel Scaffold with Multifold Bioactivity: Perspective in Bone Tissue Repair
Source: Gels. 2024 Sep 30;10(10):631. doi: 10.3390/gels10100631 (PMC11507435; doi:10.3390/gels10100631)
Supplement: Supplementary file 1 [file gels-10-00631-s001.zip › gels-3231411-supplementary.pdf]

Electronic Supplementary Information

BioMOF@cellulose glycerogel scaffold with multifold bioactivity: perspective in bone tissue repair

Albert Rosado,[a,*] Alejandro Borrás,[a] Miguel Sánchez-Soto,[b] Magdaléna Labíková,[c,d] Hubert Hettegger,[c,e] Rosa Ana Ramírez-Jiménez,[f,g] Luís Rojo,[f,g] Luís García-Fernández,[f,g] María Rosa Aguilar,[f,g] Falk Liebner,[c] Ana M. López-Periago,[a] José A. Ayllón,[h,*] Concepción Domingo[a,*]

[a] Institut de Ciència de Materials de Barcelona (ICMAB), Consejo Superior de Investigaciones Científicas (CSIC), Campus UAB s/n, 08193 Bellaterra, Spain

E-mail: arosado@icmab.es, conchi@icmab.es

[b] Departament de Ciència i Enginyeria de Materials, Escola d'Enginyeria de Barcelona Est. (EEBE), Universitat Politècnica de Catalunya-Barcelona Tech (UPC), 08019 Barcelona, Spain

[c] Institute of Chemistry of Renewable Resources, University of Natural Resources and Life Sciences, Vienna (BOKU), Konrad-Lorenz-Strasse 24, A-3430 Tulln an der Donau, Austria

[d] Department of Organic Chemistry, University of Chemistry and Technology, Prague (UCT), Technická 5, 160 00 Praha 6-Dejvice, Czech Republic

[e] Christian Doppler Laboratory for Cellulose High-Tech Materials, University of Natural Resources and Life Sciences, Vienna (BOKU), Konrad-Lorenz-Strasse 24, A-3430 Tulln an der Donau, Austria

[f] Instituto de Ciencia y Tecnología de Polímeros (ICTP-CSIC), C/Juan de la Cierva, 3, 28006 Madrid, Spain

[g] Networking Biomedical Research Centre in Bioengineering, Biomaterials and Nanomedicine (CIBER-BBN), Av. Monforte de Lemos, 3-5, 28029 Madrid, Spain

[h] Departament de Química, Universitat Autònoma de Barcelona (UAB), Campus UAB s/n, 08193 Bellaterra, Spain. E-mail: JoseAntonio.Ayllon@uab.es

Table of contents

Figures

Figure S1. ^{1}H-NMR of CaSyr-1(ibu) after HF treatment and filtration 3

Figure S2. TEM image of net NCC 4

Figure S3. Compressive stress-strain curve for CaSyr-1(ibu)@NCC-G 5

Figure S4. Drug release data fitted with the Korsmeyer-Peppas model 6

Figure S5. Optical image of glycerol drops in CaSyr-1@NCC-G leachate 7

Figure S6. Retention times for ibu, H_2Syr and DNBA 8

Figure S7. Calibration curves for HPLC measurements 9

Figures

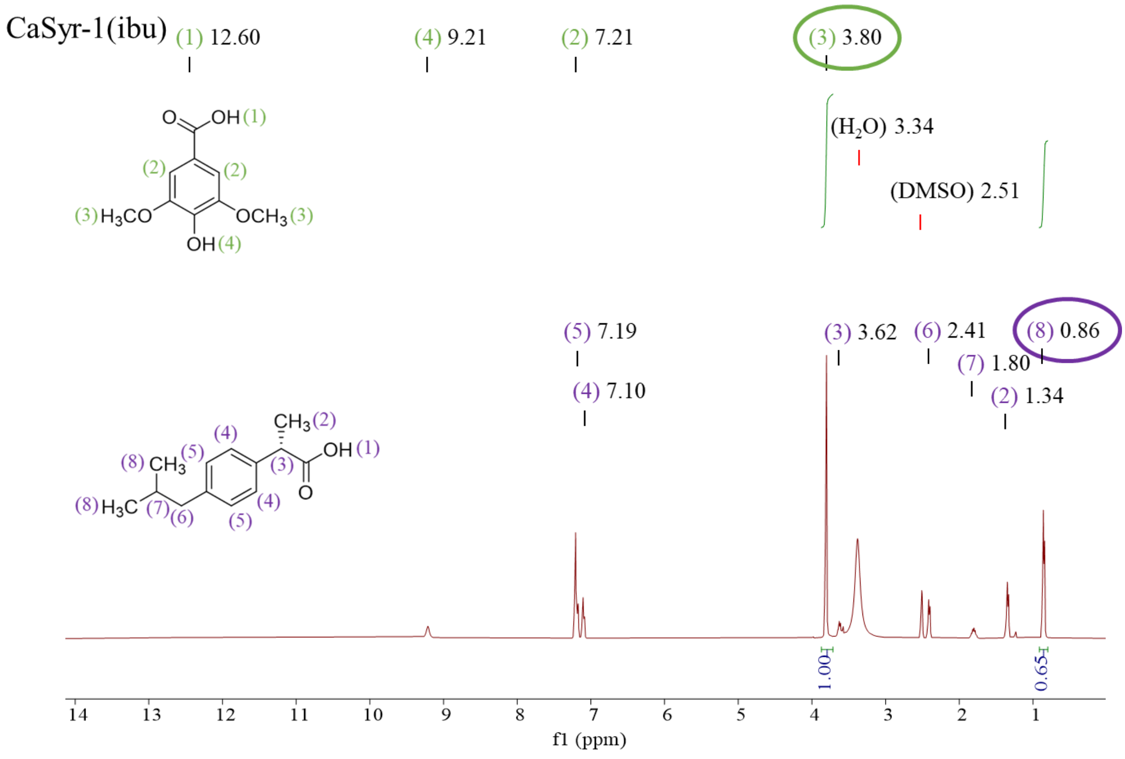

Figure S1. ^{1}H-NMR of CaSyr-1(ibu) after hydrofluoric acid digestion and filtration.

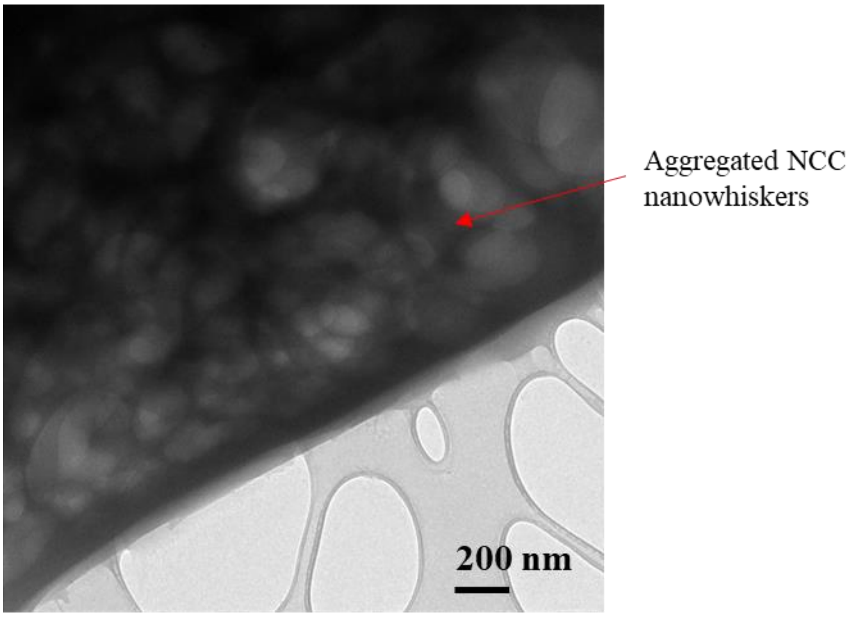

Figure S2. TEM image of net NCC.

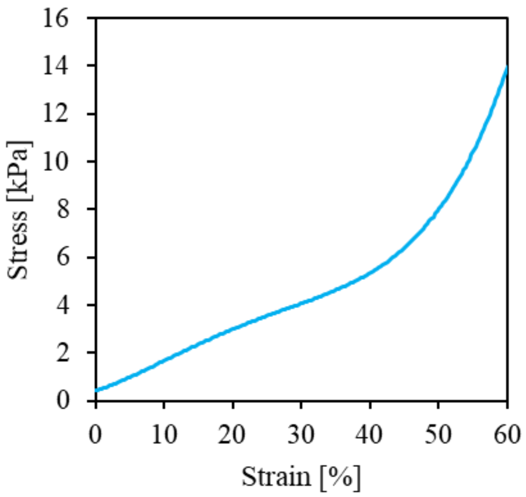

Figure S3. Compressive stress-strain curve for the CaSyr-1(ibu)@NCC-G glycerogel.

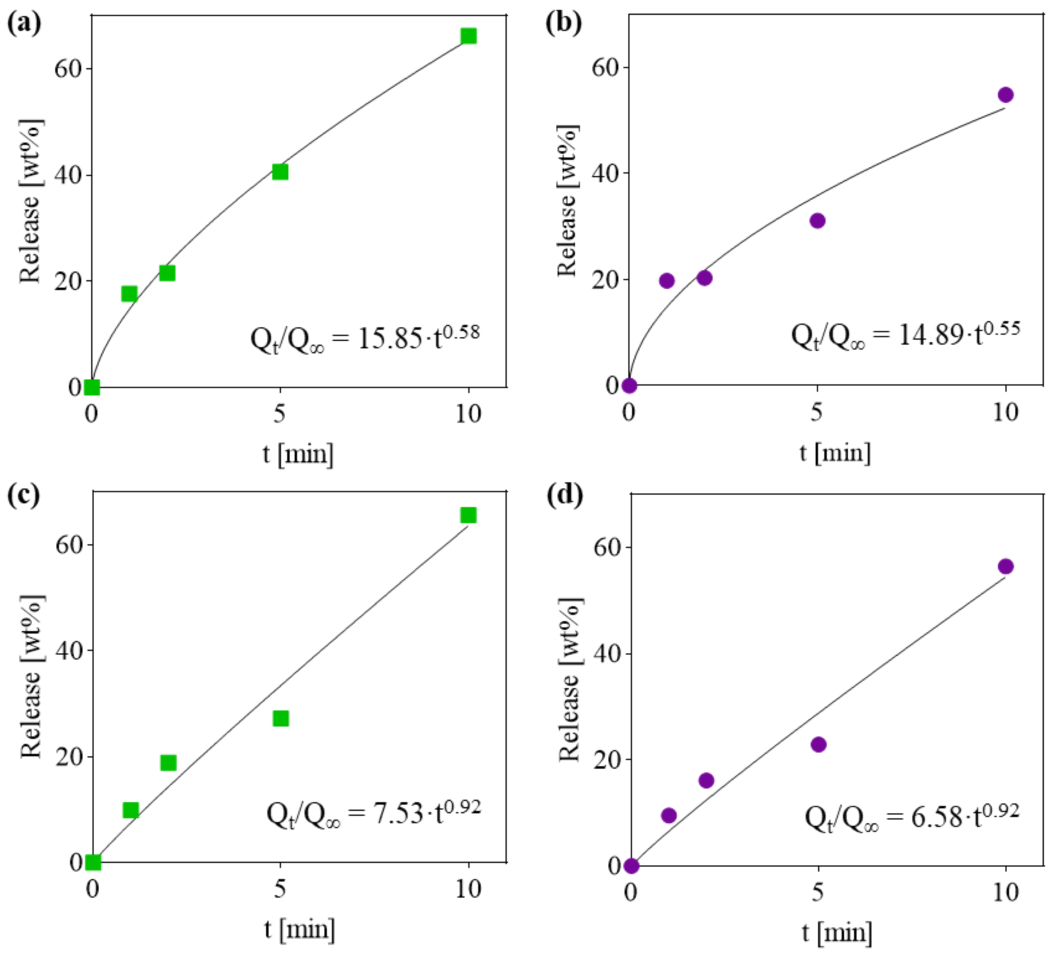

Figure S4. Points up to *ca.* 60% drug release data, in PBS at 310 K, fitted with the Korsmeyer-Peppas kinetic model $Q_t/Q_\infty = kt^n$ for: (a) syringic acid and (b) ibuprofen in CaSyr-1(ibu) NPs, and (c) syringic acid and (d) ibuprofen in CaSyr-1(ibu)@NCC-G glycerogel.

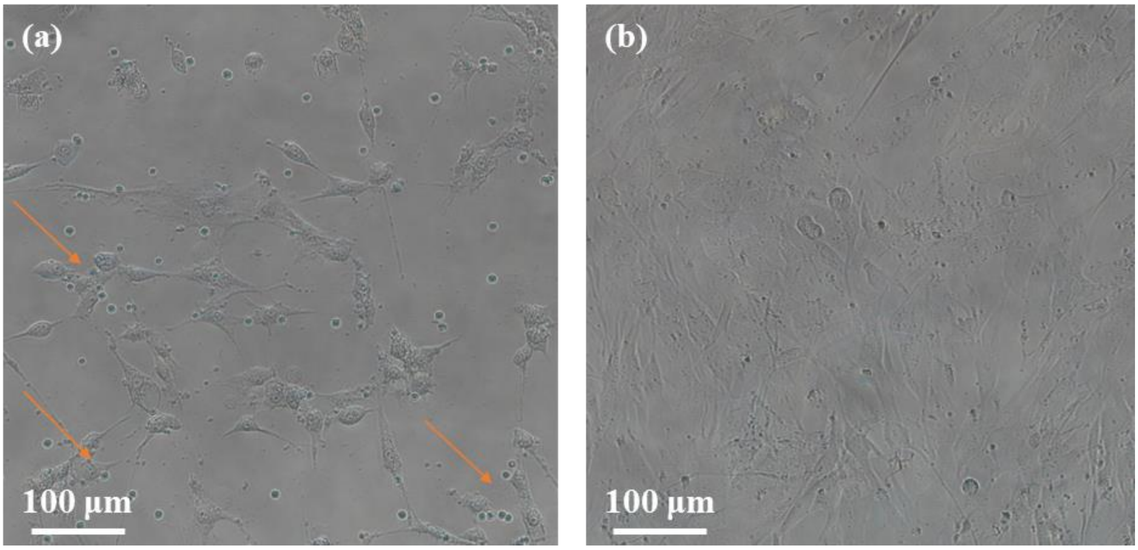

Figure S5. Optical image of lixiviates taken after 24 h from: (a) CaSyr-1@NCC-G, showing the birefringent glycerol drops, and (b) control sample.

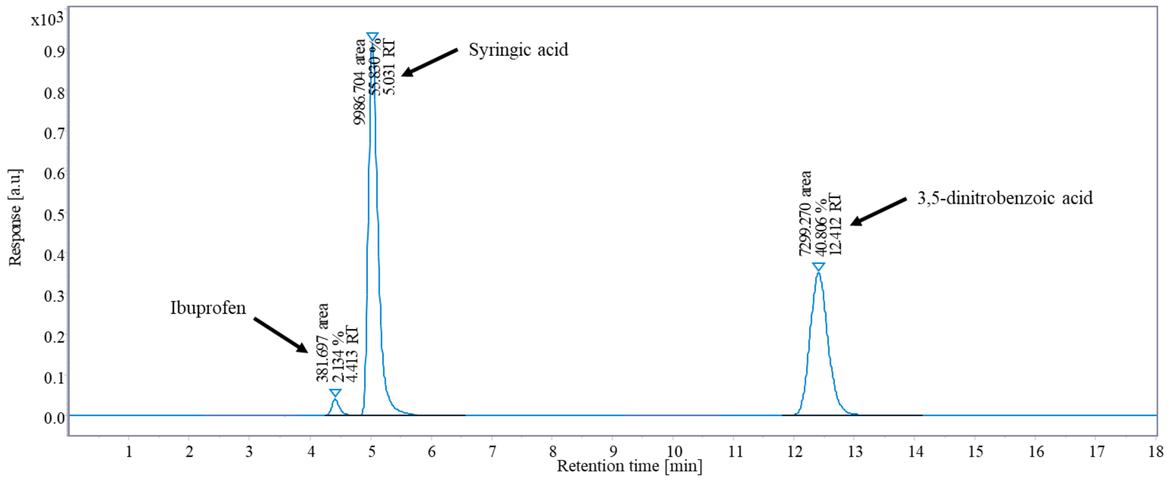

Figure S6. HPLC retention times for ibu, H_2Syr and DNBA.

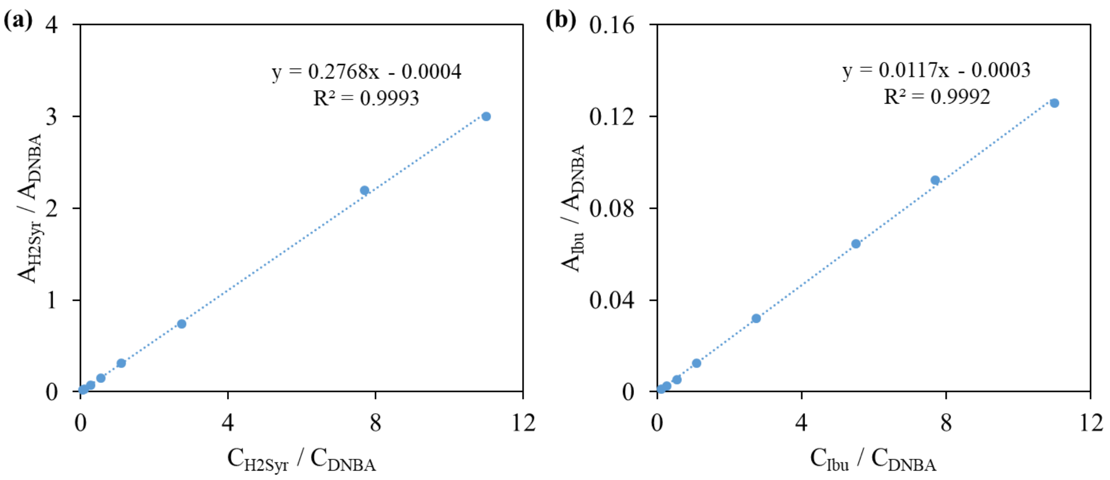

Figure S7. Calibration curves, performed in PBS at 310 K in the presence of DNBA as internal standard, for HPLC measurements of: (a) syringic acid and (b) ibuprofen.
